# The Reg3α (HIP/PAP) Lectin Suppresses Extracellular Oxidative Stress in a Murine Model of Acute Liver Failure

**DOI:** 10.1371/journal.pone.0125584

**Published:** 2015-05-04

**Authors:** Nicolas Moniaux, Marion Darnaud, Kévin Garbin, Alexandre Dos Santos, Catherine Guettier, Didier Samuel, Gilles Amouyal, Paul Amouyal, Christian Bréchot, Jamila Faivre

**Affiliations:** 1 INSERM, U1193, Centre Hépatobiliaire, Villejuif, F-94800, France; 2 Université Paris-Sud, Faculté de Médecine, Villejuif, F-94800, France; 3 Assistance Publique-Hôpitaux de Paris (APHP), Hôpital Universitaire Paul Brousse, Villejuif, F-94800, France; 4 Alfact Innovation, Paris, France; Faculty of Medicine & Health Sciences, UNITED ARAB EMIRATES

## Abstract

**Background and Aims:**

Acute liver failure (ALF) is a rapidly progressive heterogeneous illness with high mortality rate and no widely accessible cure. A promising drug candidate according to previous preclinical studies is the Reg3α (or HIP/PAP) lectin, which alleviates ALF through its free-radical scavenging activity. Here we study the therapeutic targets of Reg3α in order to gain information on the nature of the oxidative stress associated with ALF.

**Methods:**

Primary hepatocytes stressed with the reactive oxygen species (ROS) inducers TNFα and H_2_O_2_ were incubated with a recombinant Reg3α protein. ALF was induced in C57BL/6J mice by an anti-CD95 antibody. Livers and primary hepatocytes were harvested for deoxycholate separation of cellular and extracellular fractions, immunostaining, immunoprecipitation and malondialdehyde assays. Fibrin deposition was studied by immunofluorescence in frozen liver explants from patients with ALF.

**Results:**

Fibrin deposition occurs during experimental and clinical acute liver injuries. Reg3α bound the resulting transient fibrin network, accumulated in the inflammatory extracellular matrix (ECM), greatly reduced extracellular ROS levels, and improved cell viability. Hepatocyte treatment with ligands of death receptors, e.g. TNFα and Fas, resulted in a twofold increase of malondialdehyde (MDA) level in the deoxycholate-insoluble fractions. Reg3α treatment maintained MDA at a level similar to control cells and thereby increased hepatocyte survival by 35%. No antioxidant effect of Reg3α was noted in the deoxycholate-soluble fractions. Preventing fibrin network formation with heparin suppressed the prosurvival effect of Reg3α.

**Conclusions:**

Reg3α is an ECM-targeted ROS scavenger that binds the fibrin scaffold resulting from hepatocyte death during ALF. ECM alteration is an important pathogenic factor of ALF and a relevant target for pharmacotherapy.

## Introduction

Acute liver failure (ALF) is a rapidly progressive illness of diverse etiologies characterized by a massive cell death and a suppression of the regenerative capacity of the liver, often leading to multi-organ failure [1–3]. Over the last decades, progress in patient care has mostly concerned intensive care unit protocols and emergency liver transplantation programs [4,5]. No pharmaceutical drugs against ALF are available, except for acetaminophen-induced ALF, which is treatable by a specific antidote, N-acetylcysteine (NAC). The mechanisms of liver cell injury in ALF are etiology-dependent and involve multiple interconnected death pathways, making it difficult to identify pertinent therapeutic targets. Hepatocellular oxidative cell death, which is a shared process among different ALFs, is not well understood, except, to some extent, in the case of acetaminophen-induced hepatotoxicity [6]. There is indirect evidence that ALF, like other acute tissue injuries, activates a specific fibrogenesis, different from the common fibrosis of chronic liver diseases [7–10]. It is widely recognized that the extracellular matrix (ECM) contains structural and functional proteins that play a key role in the regulation of cellular function, organization and behavior [11,12]. For example, the ECM glycoprotein fibronectin has recently been claimed to be a crucial prosurvival factor in ALF [13]. However, extracellular materials are generally ill protected against oxidation for lack of antioxidants and repair enzymes [14,15]. It can therefore be conjectured that the ECM components of liver cells are strongly oxidized and thus structurally and functionally impaired during ALF. If so, reinforcing the antioxidant arsenal of the ECM could constitute a new clue guiding the search for anti-ALF drugs. Here we substantiate this view by demonstrating that the anti-ALF potential of the drug candidate Reg3α (also known as HIP/PAP) is based on its capability of restoring the functionality of the altered ECM. Reg3α is a secreted C-type lectin consisting of a single 16-kDa carbohydrate recognition domain and a NH2-terminal secretion signal. It is part of the intestinal innate immune system, its interaction with peptidoglycan carbohydrates allowing it to bind and kill Gram-positive bacteria [16–18]. Moreover, Reg3α is an anti-inflammatory and survival factor in a variety of eukaryotic cells [19–25] thanks, among other things, to its reactive oxygen species (ROS) scavenging activity [26].

## Materials and Methods

### Study approval

Research using human tissues was approved by the review board of the Centre Hépatobiliaire (Paul-Brousse Hospital, Villejuif). All patients provided written informed consent prior to the analysis of the retrieved tissues. Human tissue samples were provided by the Biological Resource Centre of the Paris-Sud Faculty of Medicine, Villejuif, France (Approval Number: 2011/39938). Fresh human tissues were obtained after surgical resection by the Pathological Department of the Centre Hépatobiliaire (Paul-Brousse Hospital, Villejuif). Tissues were cut in cubes, put in cryomolds prior to be covered with cryogel and snap frozen in isopenthane/drye ice (-120°C), and finally stock at -80°C until processing. Freezing time after liver resection was about of 30 min when surgery was done at day time, but could go up to 5 hours when surgery was process at night. Control and ALF samples were processed similarly. The biological and pathological features of patients involved in this study are summarized in Table 1. Animal studies were performed in compliance with the institutional and European Union guidelines for laboratory animal care and approved by the institutional ethics committee (Comité d’Ethique en Experimentation Animal Campus CNRS Orléans C2EA-3).

**Table 1:** Clinical characteristics of patients involved in the study.

Patient	Sex	Age	Disease	Etiology	Diagnostic	ASAT UI/l	ALAT UI/l	TP %	Time days	Management
1	Female	29	ALF	Acetaminophen	blood analysis	3575	2781	19	6	MARS:NAC
2	Female	58	ALF	Acetaminophen	FNA	7730	7158	10	2	MARS:NAC
3	Female	38	ALF	Acetaminophen	FNA	515	626	9	6	MAR-S/NAC
4	Female	18	ALF	Acetaminophen	blood analysis	2318	4180	15	6	MARS:NAC
5	Female	17	ALF	Drug	FNA	76	461	24	6	NAC
6	Female	34	ALF	Drug	FNA	561	589	12	10	
7	Female	69	ALF	Mushroom	blood analysis	2913	4387	21	7	MARS
8	Female	37	ALF	HBV	Serologic marker	235	1375	11	5	MARS/NAC
9	Female	27	ALF	HBV	Serologic marker	1530	3020	10	5	NAC
10	Female	68	ALF	Autoimmune	FNA	762	745	17	29	Vitamin K/Antibiotics
11	Female	65	ALF	Autoimmune	blood analysis	137	110	20	37	MARS
12	Female	28	ALF	Autoimmune	FNA	28	1751	8	8	
13	Male	41	ALF	Undetermined	blood analysis	6481	4011	18	3	Antibiotics
14	Female	39	ALF	Undetermined	FNA	953	631	19	16	MARS/NAC
15	Female	57	ALF	Undetermined	FNA	823	687	23	30	
16	Female	27	ALF	Undetermined	FNA	269	154	20	69	MARS
17	Female	31	ALF	Undetermined	FNA	1021	1072	16	90	MARS
18	Male	68	AN			26	25	81		
19	Female	68	AN			68	85	87		
20	Male	61	AN			24	29	100		

ALAT, **Alanine Aminotransferase;** ALF, Acute Liver Failure; AN, Amyloid neuropathy; ASAT, Aspartate Aminotransferase; FNA, Fine Needle Aspiration; MARS, Molecular Adsorbent Recirculating System; NAC, N-acetylcysteine; TP, Prothrombin level; Time, time in days from diagnostic to liver transplantation; UI, International Unit.

### Recombinant human Reg3α protein

The Reg3α protein was produced in Escherichia coli, purified to ≥99% and released in batches in compliance with the clinical grade manufacturing process by PX'Therapeutics. It corresponds to the addition of one amino-terminal methionine to the sequence of the secreted (i.e., lacking the 26–amino acid signal sequence) form of the human endogenous Reg3α or HIP/PAP (NP_620355).

### Primary hepatocytes

Human hepatocytes were prepared from a lobectomy segment following surgical liver resection in patients with colorectal cancer metastases. Murine hepatocytes were retrieved from 6-week-old male C57Bl/6J mice (Charles River, L'Arbresle Cedex, France). Hepatocyte were isolated following a two-step collagenase method [27] and platted in William’s medium (Life Technologies, Saint Aubin, France) containing 10% calf serum without collagen coating. Two days later, cellular death was induced with TNFα/Actinomycin D (20 ng/mL/50 ng/mL) maintained throughout the cell culture or with a 1 mM H_2_O_2_ pretreatment for 1 hour. Cell viability studies were performed under the condition that the cell death rate in stressed control cultures was of 55±10%, a condition easier to fulfill without collagen coating. Hepatocytes were stimulated with death inducers and 0.24 μM Reg3α or an equivalent volume of buffer (vehicle). Cells were fixed and stained with 5 μg/mL of Hoechst 33342 (Cell Signaling, Ozyme, France). Living and apoptotic cells were quantified using a Zeiss Axio Imager.M1. Cell quantification was performed on 20 random microscopic fields per sample. The normalized cell viability is defined as (n_Reg3_-n_Veh_)/(n_Bas_-n_Veh_), where n_Reg3_, n_Veh_ and n_Bas_ are the numbers of living cells in Reg3α-treated, vehicle-treated and non-intoxicated (basal) cultures at 24h post stress, respectively. Collagenase type IV, mouse recombinant TNFα, Actinomycin D, H_2_O_2_ solution 30%, Heparin, and MG132 were purchased from Sigma Aldrich.

### Murine model of ALF

Six-week-old C57BL/6J mice were maintained for 1 week at 22°C before experiments. ALF was induced by intravenous injection of an anti-CD95 Fas antibody (BD Pharmigen, Le Pont de Claix, France) at 170 μg.kg^-1^, which is a lethal dose for about 60% of the mice within 24 hours. Reg3α was intravenously injected at the dose of 2.5 mg/kg. The control groups were injected with equivalent volumes of vehicle. Mice were sacrificed by decerebration and livers harvested for deoxycholate separation of cellular and extracellular fractions, immunostaining and malondialdehyde assays.

### Real-time qPCR

Total RNA was isolated using TRI reagent (Sigma) according to the manufacturer’s instructions. Reverse transcription was performed using oligo(dT) primer and 1μg of total RNA in a final volume of 20 μL with the RevertAid First Strand cDNA Synthesis Kit (Thermo scientific). Real time qPCR was performed using the FastStart Essential DNA green Master kit (Roche) on a lightcycler Instrument equipped with the SW 1.1 data analysis software (Roche). Analyses were done in triplicate. Data were normalized using HPRT as housekeeping gene. Primer sequences are: Reg3α forward: 5’-TGGATTGGGCTCCATGACCC-3’OH, Reg3α reverse: 5’-TTCGGGATGTTTGCTGTCTG-3’OH, HPRT forward: 5’-AGGACCTCTCGAAGTGT-3’OH, HPRT reverse: 5’-TCAAATCCCTGAAGTACTCAT-3’OH.

### Immunoblotting

Primary hepatocyte cultures were washed twice with ice cold phosphate buffered saline (PBS) and then lysed in a deoxycholate (DOC) lysis buffer containing 2% sodium deoxycholate, protease inhibitors (cOmplete Protease Inhibitor Cocktail, Roche, Meylan, France), 20 mM Tris-HCl pH 8.8, 2 mM EDTA, 2 mM iodoacetamide and 2 mM N-ethylmaleimide. The DOC-soluble fractions contain the cellular material and extracellular components that are not incorporated into the ECM, whereas the DOC-insoluble pellet contains the ECM. Homogenates were passed three times through a 26-gauge needle, and centrifuged at 10,000 g for 10 min at 4°C. DOC-insoluble fractions were solubilized in SDS lysis buffer containing 1% SDS sodium dodecyl sulphate, 20 mM Tris-HCl pH 8.8, 2 mM EDTA, protease inhibitors, 2 mM iodoacetamide and 2 mM N-ethylmaleimide. Frozen liver samples were processed in the same way. Protein concentrations were determined with the Bio-Rad Protein Assay Kit using bovine serum albumin as a standard. Aliquots of 30 μg were denaturated by boiling in Tris-Glycine SDS Buffer (Invitrogen), separated by 12% SDS-PAGE and transferred onto nitrocellulose membranes (Whatman, Dominique Dutscher, Brumath cedex, France) by electroblotting. Membranes were blocked in 5% non-fat dry milk in 0.1% Tween 20 Tris-buffered saline for 1h and probed with primary antibodies against Fibronectin (Santa Cruz Biotechnology), Fibrinogen (Dako), Actin (Santa Cruz Biotechnology, Heidelberg, Germany), mouse Reg3β (R&D Systems, Lille cedex, France), human Reg3α (Santa Cruz Biotechnology) and a lab-made human Reg3α consisting of a rabbit polyclonal antibody raised against the complete Reg3α protein purified by affinity chromatography [14].

### Immunoprecipitation

Aliquots of 50 μg of proteins from DOC-soluble and insoluble fractions were diluted 1 to 10 in 1%-Triton X-100 nondenaturing lysis buffer (50 mM Tris-HCl pH 7.4, 300 mM NaCl, 5 mM EDTA, 10 mM iodoacetamide, protease inhibitors) and pre-cleared with 20 μL of protein G magnetic beads (PureProteome, Millipore, Molsheim, France) for 2 hours at room temperature. Clarified lysates were incubated with 1 μg of Fibrinogen antibody for 1h at 4°C. The immune complexes were captured using 20 μL of protein G magnetic beads at 4°C for 30min, purified on magnetic rack, washed four times in 0.1%-Triton X-100 buffer and once in ice cold PBS. Immune complexes were eluted from magnetic beads in 1X Laemmli loading sample buffer and processed for anti-Reg3α immunoblotting.

### Immunostaining

Hepatocytes grown on glass coverslip and 4 μm-thick frozen liver sections were fixed in formalin solution (10%, buffered) supplemented with 2% acetic acid for 30 min at room temperature, which is known to extract fibrinogen, fibrinogen and fibrin degradation product and non-cross-linked fibrin, thus ensuring that cross-linked fibrin is the major immunoreactant [28]. After fixation, the samples were labeled with primary antibodies (see above) for 45 min at 37°C and then with secondary (Alexa 488 donkey anti-goat, Alexa 594 donkey anti-rabbit; Life Technologies) antibodies for 30 min at 37°C. Nuclear DNA was stained with 5 μg/mL Hoechst 33342. Histopathology investigations were performed by microscopic examination of hematoxylin/eosin-stained sections of paraffin-embedded tissues.

### Lipid peroxidation

Malondialdehyde (MDA) was dosed using the thiobarbituric acid method as previously described [26]. A standard curve was prepared using the Malondialdehyde Tetrabutylammonium Salt (Sigma Aldricht, Lyon, France). The results were normalized in relation to the total protein content.

### Protein carbonylation

Carbonyl groups of 1 μg of DOC insoluble and 10 μg of DOC soluble proteins were derivatized to 2, 4-dinitrophenylhydrozone (DNP) by reaction with 2,4-dinitrophenylhydrazine in solution in 2N HCl, incubated 30 min in the dark at room temperature before being precipitated by addition of an equal volume of 20% trichloroacetic acid. Protein pellets were washed four times with 1 mL of ethanol/ethyl acetate (1:1), and resuspended in SDS loading buffer. One half of each sample was analyzed by Western blot using a rabbit anti-DNP antibody while the other half was resolved on a 10% acrylamide gel for Coomassie blue staining. Amount of carbonylated and total proteins were determined by densitometry using the Image J image processing and analyzing software; results were presented as the ratio of carbonylated on total proteins.

### Statistical analysis

Two-tailed Student’s t-test was used to assess statistical differences between groups using StatView 5.0 freeware (SAS Institute, Inc, Cary, North Carolina, USA) and differences with *P*<0.05 were considered significant. All data are presented as mean over several independent experiments ± SEM.

## Results

### The Reg3α lectin binds the extracellular fibrin network of primary human hepatocytes

**Fig 1:**
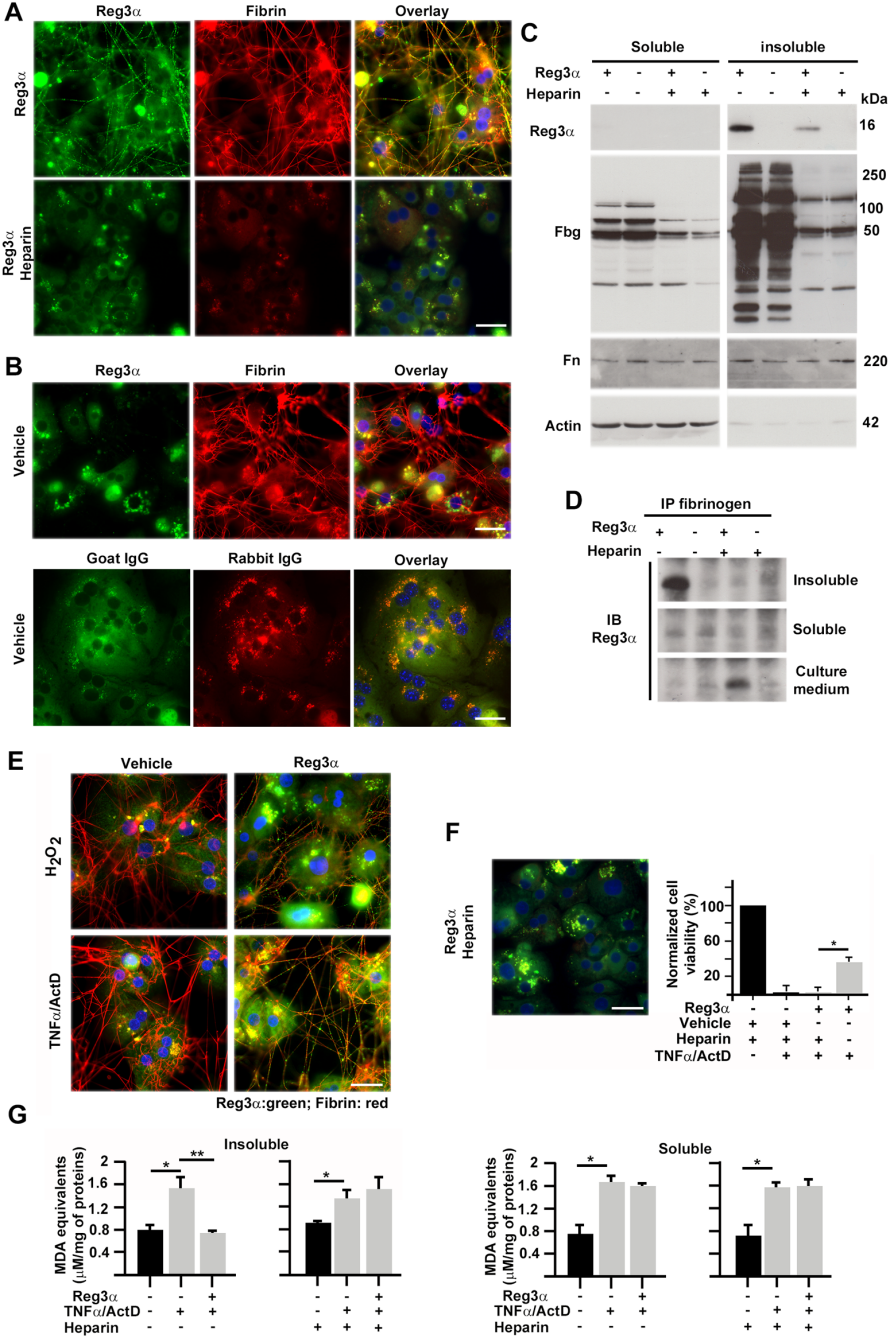
Reg3α fights oxidative stress to extracellular matrix *in vitro*. (A and B) Immunofluorescence of primary human hepatocytes (PHHs) incubated with either a recombinant Reg3α without (A, top) or with heparin (A, bottom), or (B) vehicle. DNA: blue. (C) Immunoblotting for Reg3α, fibrinogen (Fbg), and fibronectin (Fn) in DOC-soluble and insoluble extracts from PHHs cultured under the indicated conditions. (D) Anti-Reg3α immunoblotting (IB) in the indicated fractions of lysates immunoprecipitated (IP) with an anti-fibrin/fibrinogen antibody. (E) Immunofluorescence of PHHs stressed with H_2_O_2_ or TNFα/ActD and treated either with Reg3α or vehicle. (F) Left: Immunofluorescence of PHHs incubated with heparin and then TNFα/ActD and Reg3α. Right: Normalized cell viability. (G) Normalized concentration of malondialdehyde (MDA) in DOC-insoluble and DOC-soluble fractions of PHHs subjected to the same treatments as in (F). Scale bars: 50 μm. Student’s t-test, *P < 0.05; **P <0.01.

We cultured primary human hepatocytes (PHHs), incubated them with 0.24 μM of a recombinant Reg3α protein for 6h and then analyzed Reg3α subcellular distribution by immunofluorescence. Cultured PHHs synthetized an abundant extracellular matrix (ECM) of polymerized fibrin and fibronectin (Fig 1A and S1A Fig), which covered the entire cell layer 48h after platting. A similar deposition of a fibrillar matrix in rat hepatocyte cultures was previously reported [29]. This ECM contained two different fiber networks, one lying on top of the cells and the other, made of thinner fibers, on the substratum. The Reg3α staining mostly appeared in the form of small clusters lining the top fibrin-fibronectin network and scattered throughout the substratum (Fig 1A, S1A and S1B Fig). Incubations performed with fluorescein isothiocyanate (FITC)-coupled Reg3α yielded a similar Reg3α staining pattern (S1C Fig). We detected some intracellular staining with the Reg3α antibody in the PHHs cultured with vehicle (Fig 1B, upper row), which was however essentially nonspecific (Fig 1B, lower row), indicating that the exogenous Reg3α was not taken up by PHHs. We nevertheless wondered whether a fast proteolysis of a taken-up Reg3α had not occurred. A pre-treatment with 10 μM of the proteasome inhibitor MG132 did not increase Reg3α signal in the soluble fractions of PHHs, discarding this possibility (S2 Fig). An addition of 50 μg/mL of heparin prevented both extracellular fiber network formation and Reg3α accumulation in the PHH cultures, demonstrating that the latter was dependent on the former (Fig 1A and S1A Fig). Deoxycholate-insoluble ECM-enriched fractions and their correlative soluble cellular fractions were extracted from PHHs cultured either with or without Reg3α. The anti-fibrinogen immunoblotting revealed the presence of high molecular weight (MW) fibrin-fibrinogen complexes and low MW fibrin degradation products in the insoluble fractions (Fig 1C and S2 Fig). These fractions displayed a strong 16-kDa Reg3α signal, which was inexistent or weak in the soluble fractions. When the PHHs were treated with heparin, the insoluble fractions displayed a much-reduced Reg3α signal, confirming that the binding sites for Reg3α were located on the fibrin fibers. Non-denaturing gel electrophoresis showed that Reg3α and fibrin displayed comparable electrophoretic mobility in the insoluble fractions, suggesting that these proteins co-migrated and thus were bound together by a protein-protein interaction (S3 Fig). Proteins from insoluble and soluble fractions incubated, or not, with Reg3α and heparin were immunoprecipitated with an anti-fibrinogen antibody and then analyzed by anti-Reg3α immunoblotting. A strong Reg3α signal was found in the insoluble fraction of the PHHs, confirming a protein-protein interaction between Reg3α and fibrin (Fig 1D). A relatively strong Reg3α signal was also found in the culture medium of PHHs with heparin, but not without heparin. This is attributable to the release into the culture medium of residual fibrin fragments interacting with Reg3α following addition of heparin. In other words, circulating fibrins can interact with, and serve as carriers for Reg3α. We also monitored the distribution of Reg3α in PHHs stressed with the ROS-inducers TNFα [30] and H_2_O_2_. In the two conditions, a fiber network scattered with Reg3α was observed as early as 2h post incubation (Fig 1E). At longer times post incubation, Reg3α coalesced into large clusters (S1D Fig).

### Reg3α reduces oxidative damage to the extracellular matrix

We studied Reg3α-mediated liver cell viability as a function of the presence, or absence, of an extracellular fibrin network. Adding Reg3α to TNFα-stressed PHH cultures resulted in a substantial increase in cell viability, as previously shown [26]. By contrast, an addition of heparin at the time of cell platting prevented the formation of a fibrin network and totally suppressed the Reg3α-mediated PHH survival gain (Fig 1F). When heparin was added several hours post platting, the fibrin network was not altered and the cell survival gain was preserved (S4 Fig). This underscores the dependence of the cytoprotective effect of Reg3α upon the production of an extracellular fibrin network. Next we measured the concentration of malondialdehyde (MDA), an end product of ROS-mediated lipid peroxidation, in the insoluble and soluble fractions of damaged PHHs in the presence, or the absence, of Reg3α and heparin. Stressing PHHs with TNFα resulted in a significant (about twofold) increase in MDA level in both the soluble and insoluble fractions. A treatment with Reg3α suppressed the TNFα-induced increase in MDA level in the insoluble fractions of PHHs, and not in the soluble ones. The anti-oxidant effect of Reg3α in the insoluble fractions did not occur in PHHs cultured with heparin (Fig 1G). Globally, these results indicate that Reg3α binds the fibrin-fibrinogen network of PHHs, reduces their extracellular oxidative stress and thereby facilitates their survival in inflammatory environment.

### Reg3α is targeted to the hepatic ECM formed during Fas(CD95)-induced ALF in mice

**Fig 2:**
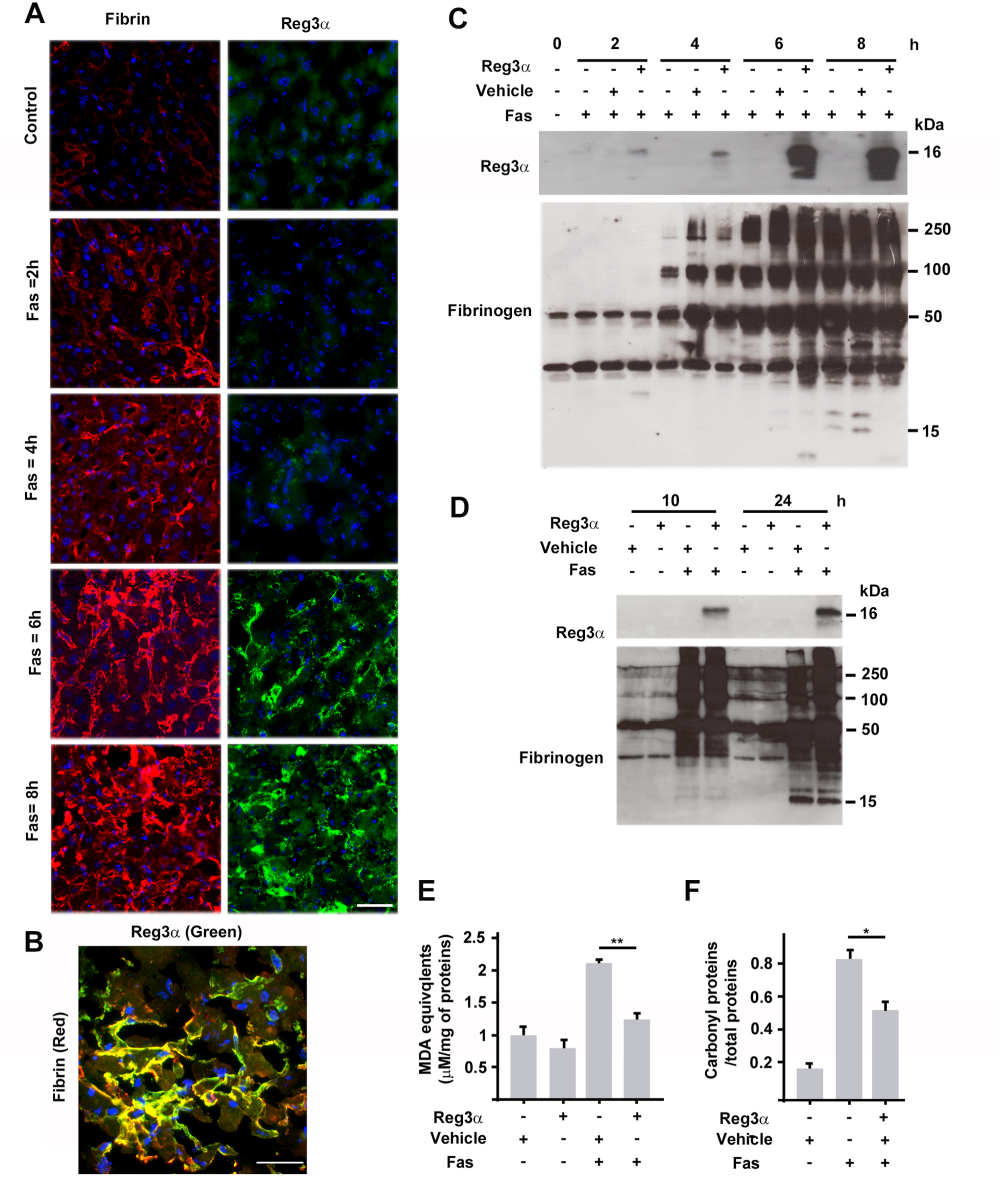
Reg3α is targeted to the provisional extracellular matrix of livers injured by the anti-CD95 (Fas) antibody. (A) Immunofluorescence of liver cryosections from mice intoxicated with Fas for the indicated duration times and injected with Reg3α 2 hours before sacrifice. DNA: blue. Control: healthy livers. (B) Fibrin-Reg3α double immunostaining at Fas = 8h. (C) Immunoblotting for Reg3α and fibrinogen in DOC-insoluble fractions of livers under the same conditions as (A). (D) Reg3α was injected 6 hours post-Fas intoxication. (E) Normalized concentration of malondialdehyde (MDA) and (F) ratio of carbonylated proteins to total proteins in DOC-insoluble fractions of livers intoxicated with Fas for 6 hours and treated with Reg3α only 30 min before sacrifice. *P < 0.05, **P < 0.01. Scale bars: 50 μm.

We studied fibrogenesis and its interaction with Reg3α in a murine model of ALF induced by the anti-CD95 antibody (Fas). This model is known to be relevant to human viral ALF with mitochondrial damage and ROS overproduction [31,32]. We first looked at whether the fibrin network developed by primary murine hepatocytes (PMHs) binds human Reg3α. Indeed, we found that Reg3α accumulated in the form of small clusters on the extracellular fibrin network in PMHs, like in PHHs (S5A Fig). Immunoblots of the insoluble, fibrin polymer-rich, fractions of PMH extracts displayed strong monomeric and polymeric Reg3α signals. These were greatly reduced by a treatment of the PMHs with heparin (S5B Fig). Next, we injected mice intravenously with a single dose of 2.5 mg/kg of Reg3α at different time points after Fas-intoxication and sacrificed them 2h after Reg3α injection. As controls, we used Fas-intoxicated mice injected with vehicle and healthy mice injected with vehicle or Reg3α. Livers were analyzed by immunofluorescence and fractions of liver extracts by immunoblotting. We found an extensive fibrin deposit in the sinusoid spaces of the livers, in agreement with previous reports on drug-induced ALF in rodents [33–36]. The fibrin deposit increased gradually as the duration of Fas-intoxication increased, and reached a substantial density from 6h post-Fas (Fig 2A and S6A Fig). In damaged livers treated with Reg3α, the Reg3α staining remained weak until 4h post-Fas and became stronger at 6h post-Fas (Fig 2A). Reg3α was co-localized with fibrin (Fig 2B). We were not able to detect Reg3β, the murine homolog of human Reg3α, in the damaged livers of Fas-intoxicated mice injected with vehicle (S6A Fig) although we found an activation of the Reg3β gene by qPCR (S6B Fig). High-MW fibrin bands were detected at 4h post-Fas in the insoluble fractions and increased in intensity over time. Reg3α was accumulated in the insoluble fractions in the same time range (Fig 2C and S7 Fig). This concomitancy suggests that Reg3α preferentially binds higher-MW fibrins and defines a time threshold for extracellular accumulation of Reg3α of about 6h in the experimental model under study. It could also explain the previous observation that an injection of Reg3α made before 6h post-Fas did not significantly improve survival and did not attenuate oxidative damage during ALF [26]. In other words, the therapeutic time threshold for Reg3α corresponded to the time at which its molecular target, namely, the inflammatory fibrin-fibrinogen matrix, appeared in the course of ALF. The extracellular accumulation of Reg3α continued at longer times during the fibrolytic process concomitant with liver repair and regeneration. A strong Reg3α band was still seen in the immunoblots of insoluble fractions at 24h post-Fas, establishing the stability of Reg3α in the remodeled ECM (Fig 2D). Finally, a single injection of Reg3α at a time when the fibrin matrix was abundant (namely, at 6 h post-Fas) sufficed to rapidly reduce the Fas-induced extracellular oxidative damage: as shown in Fig 2E, the twofold increase in hepatic MDA level due to Fas intoxication was suppressed 30 min after Reg3α injection. This result was further validated by measuring the level of extracellular carbonylated proteins. A massive increase of protein carbonylation was noticed 6 h post-Fas, this level was reduced by 40% in animals treated with Reg3α. This demonstrates that Reg3α can be an effective and fast-acting ECM-associated antioxidant *in vivo*.

### Fibrin deposition in hepatic sinusoids during acute liver failure in humans

**Fig 3:**
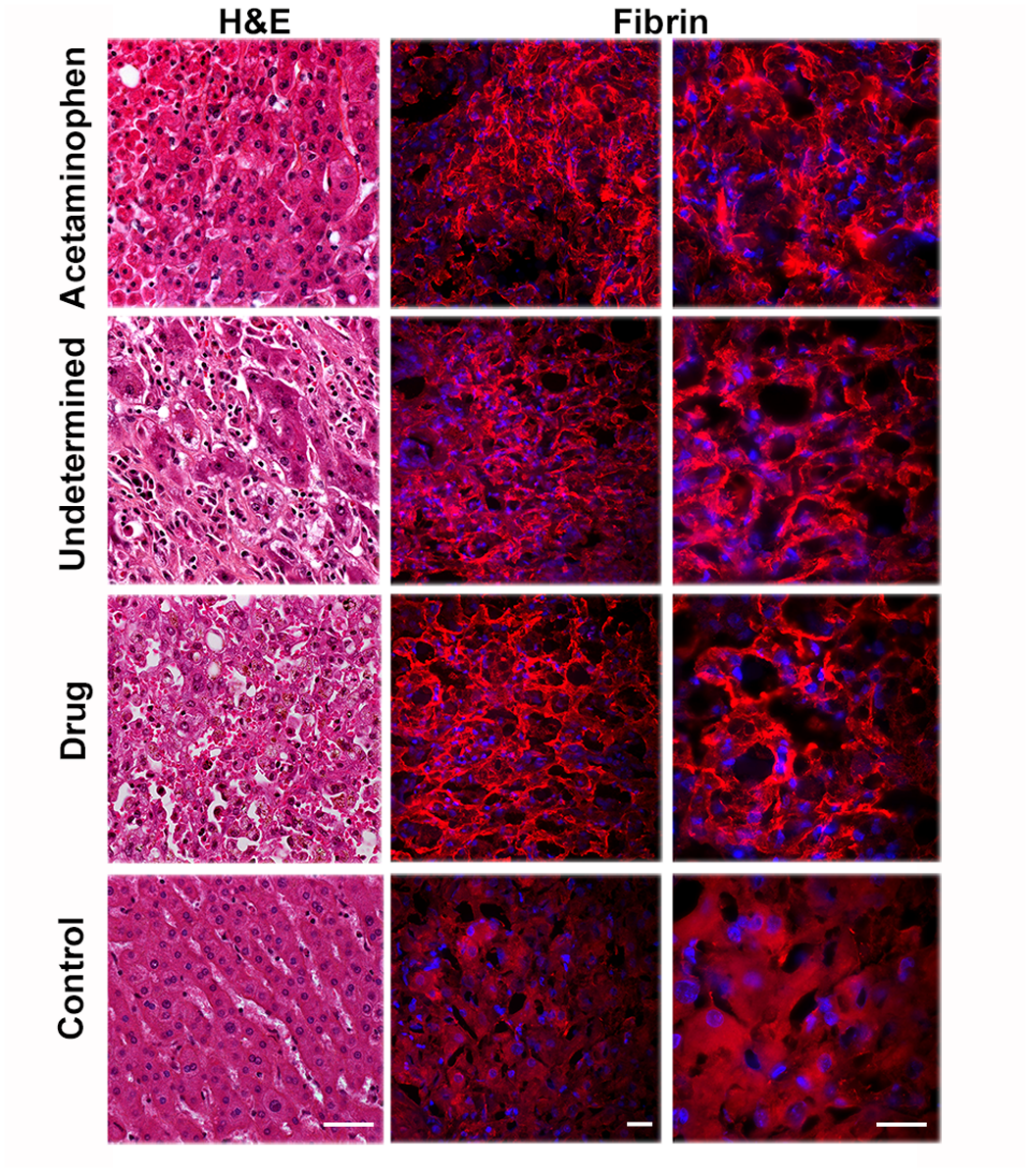
Abundant fibrin deposition in human ALFs. Histological analysis using hematoxylin/eosin (H&E) staining (left), anti-fibrin immunofluorescence (right) of sections of liver explants from patients with end stage acute liver failure of different etiologies. Control: liver explants from patients with amyloid neuropathy. Scale bars: 50 μm.

According to our findings, the existence of a fibrin-fibrinogen scaffold in the fibrosis associated with human ALF would be an essential requirement for a pharmacological action of Reg3α. This has not yet been documented, to our best knowledge. We studied fibrin deposit in frozen liver explants from 17 patients with ALF of various etiologies (4 acetaminophen-, 2 drug-, 1 mushroom-induced, 2 HBV-, 3 autoimmune-induced ALFs, and 5 ALFs of undetermined origin) by immunofluorescence. Liver explants from 3 patients with amyloid neuropathy were used as controls [37]. We found substantial fibrin deposits lining sinusoid spaces within the areas of hepatocellular necrosis in all the end-stage ALF samples studied, and not in the normal-liver ones (Fig 3) Seven explants of patients with chronic and malignant disorders were analyzed as a control test. Fibrin deposition was detected in all explants, even non-neoplastic parenchyma adjacent to tumors (S8 Fig). This supports expectations that exogenous Reg3α might accumulate on such deposits and thereby act as an ECM-targeted antioxidant in human ALF and other hepatic disorders.

## Discussion

The mechanisms of liver damage and regeneration during ALF are still ill understood. It is generally accepted that, in case of massive hepatic cytolysis, hepatocytes lose their capacity to regenerate, and a backup mechanism leading to liver stem cell proliferation/differentiation takes over. Depending on the cytolysis extension, this second mechanism does not always allow a fast enough liver repopulation, with for consequence a multi-organ failure and an urgent need for liver transplantation. During ALF, a number of molecular pathways governing inflammation and cell death are activated in concert, inducing massive tissue injury and suppressing liver cell proliferation. The oxidative stress following from a massive—in fact, so massive that it exceeds the detoxification capacity of the hepatocytes—production of reactive oxygen and nitrogen species is presumably an important factor in the pathophysiology of ALF [38]. There is experimental evidence that ROS are key executers of hepatocyte death, their generation preceding the onset of apoptosis after Fas-induced hepatocyte death [39]. Apoptosis could be prevented by a pre-treatment with the superoxide dismutase mimic (MnTBAP), either on primary culture of hepatocytes or in the Fas-induced experimental hepatitis mice model [39]. ROS modulated hepatocyte death by decreasing FLIP level and by increasing cytochrome C release in a BID-dependent manner after TNFα- and Fas-induced death receptors activation [40]. In addition, iron chelation, that prevents the transition from superoxide to hydroxyl radical by the Fenton reaction, rescued mice from Fas-induced hepatitis [41]. Currently, the unique antidote for acute liver failure is an antioxidant therapy, i.e. continuous intravenous infusion of NAC, which is recommended by the guidelines to treat patients with acetaminophen poising [42,43]. NAC is efficient to replenish mitochondrial and cytosolic glutathione within the liver and to treat the main causes of morbidity in ALF, e.g. multi-organ failure (respiratory distress syndrome) [44] and sepsis [45]. Recent studies showed that NAC could benefit to patients independently of glutathione repletion and so be efficient in drug-induced liver injury [46]. Already, reports of several prospective clinical trials present evidences for its efficacy to treat non-acetaminophen patients, improving transplant-free survival and serological biomarkers [47–49]. Despite these great promises, there is place for others antioxidant drugs in the treatment of ALF as NAC efficiency requires its administration early after the onset of ALF and appears not to improve transplant-free survival of pediatric non-acetaminophen ALF [50].

This study highlights the pathogenic role of extracellular oxidative stress during ALF and thus its relevance as a target for pharmacotherapy. This raises the question of the extracellular sources of ROS that would be involved in experimental and clinical ALFs. Contrary to a widespread opinion, the transport of ROS from intracellular to extracellular compartments is not necessarily negligible. Some ROS (such as hydrogen peroxide) can diffuse through membranes, others (such as superoxide) can translocate via carriers (such as VDAC, which is expressed at the plasma membrane, and not only in mitochondria) [51]. Furthermore, extracellular sources of ROS either originating from the matrix itself or released by necrotic cells exist. It is now clearly established that tissue injuries release damage-associated molecular pattern (DAMPs) that activate complement cascade and toll-like receptors [52], both priming macrophages NADPH oxidase and neutrophils myeloperoxidase [53,54]. Activated macrophages and neutrophils generate superoxide and hydrogen peroxide radicals, neutrophils producing also hypochlorous acid. Thus ROS are generated within the extracellular spaces in close contact with hepatocytes where they can diffuse trough cell membrane to induce cell death [55].

**Fig 4:**
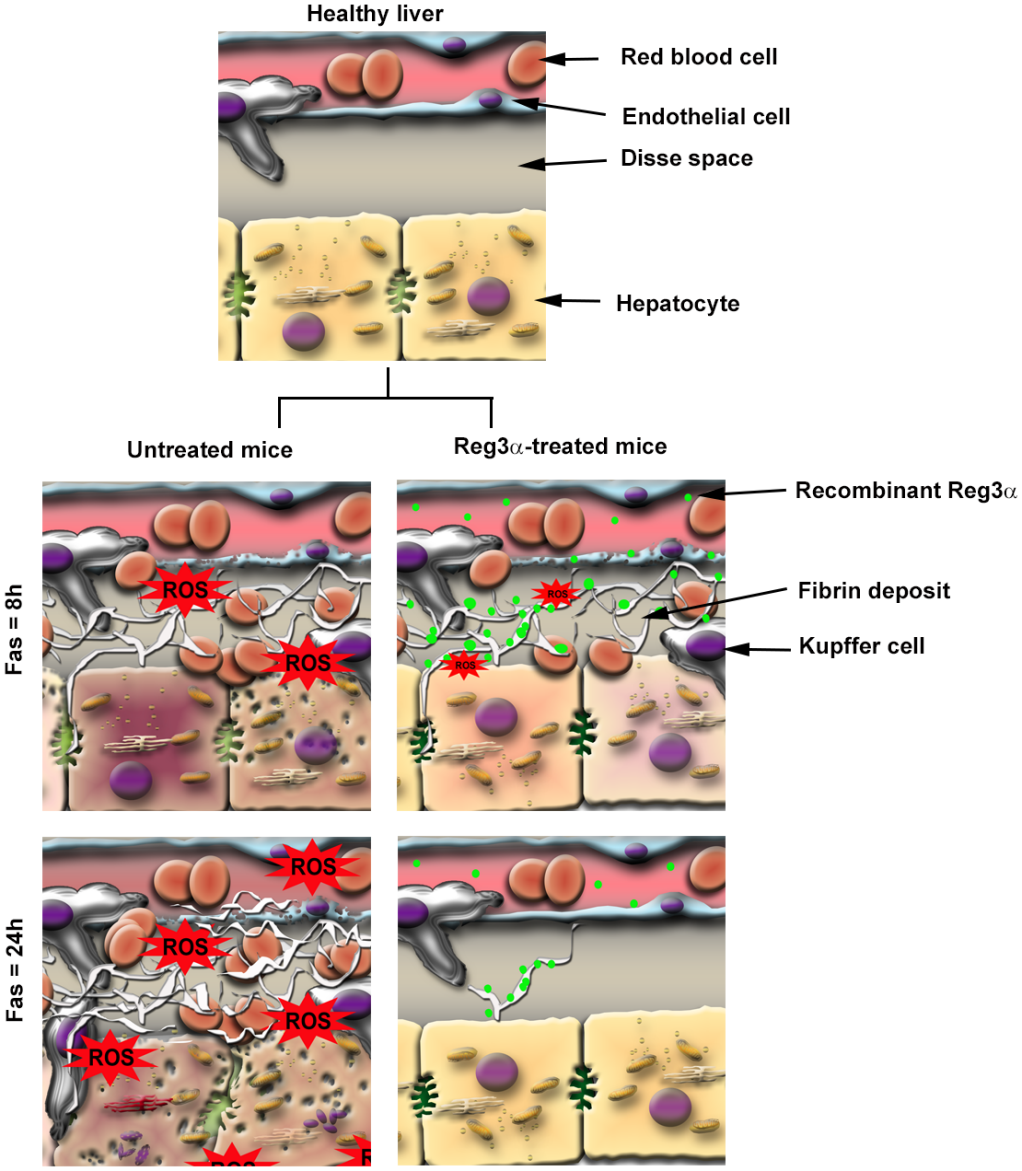
Schematic representation of the mode of action of Reg3α during Fas-induced ALF. Mice were intoxicated with the anti-CD95 antibody (Fas) and were either untreated (left) or injected intravenously with 2.5 mg/kg of Reg3α at 6h post Fas (right). Two hours later (Fas = 8h), Reg3α is accumulated on the extracellular fibrin network surrounding stressed hepatocytes, reducing the ROS concentration and improving cell survival and functionality. At 24h post intoxication, liver regeneration is under way in the Reg3α–treated mice while the fibrin network is being eliminated by physiologic fibrolysis.

It is known that ECM alteration can dramatically modify not only ECM structure but also cell fate and function. Many disorders, among which, acute and chronic liver diseases, are associated with a strong extracellular oxidative stress. However, the mechanisms connecting ECM alteration to cell behavior are poorly understood and, from the pharmacological standpoint, few drugs targeting specific ECM components are available today [56,57]. Here we used the death receptors TNF and Fas, which induce not only apoptosis, but also necrosis and ROS overproduction [58], to show that the prosurvival effect of Reg3α-HIP/PAP relied on a strong antioxidant activity specifically deployed in the stressed ECM of injured liver cells. The extracellular accumulation of Reg3α resulted from its binding to the provisional fibrin deposit that increased gradually during ALF (Fig 4). In brief, Reg3α is an ECM-targeted antioxidant that restores the functionality of ECM components involved in cell fate controlling pathways, which further studies will have to identify.

To date, liver transplantation is the only recognized therapy of proven benefit against ALF, except for acetaminophen-induced ALF, which is treatable by NAC. There are methods of liver support used while waiting for a donor liver or for the native liver to regenerate, but their effectiveness remains unproven and is currently evaluated in multicenter clinical trials [59]. Both transplantation and liver-support methods require specialized units and expensive complicated equipment. Therefore, the identification of pharmaceutical therapies would be a major step forward in the care of patients with ALF. Numerous molecules have been reported to counteract ALF by reducing hepatocyte cell death or stimulating liver regeneration during preclinical studies [2]. A few of them have entered clinical trials, among which a recombinant protein homolog of human Reg3α [26]. The selective targeting of Reg3α to ALF-related acute fibrosis demonstrated in this study constitutes a pharmacological advantage in terms of both safety and efficacy. From a therapeutic viewpoint and based on our results, a double-blinded randomized phase 2 clinical trial was conducted on patient with non-acetaminophen acute liver failure, trial that showed significant recovery induced by Reg3α in particular in patients suffering from autoimmune- and HBV-related acute on chronic liver failure [60]. Finally, it should be noted that, while the currently explored medical applications of Reg3α are focused on non-acetaminophen-related ALFs, this study suggests that they could conceivably be extended to other hepatic or non-hepatic inflammatory diseases characterized by an important extracellular fibrin deposition.

## Supporting Information

S1 Fig Reg3α colocalizes with the fibrin-fibronectin network of primary human hepatocytes. (*A-C*) Primary human hepatocytes (PHHs) were incubated with 0.24 μM of a recombinant Reg3α for 6h either without or with 50 μg/mL of heparin. Control cells were treated with an equivalent volume of vehicle. (*A*) Immunofluorescence of Reg3α-treated PHHs either without (middle) or with heparin (bottom). Reg3α: red; Fibrinonectin: green; DNA: blue. Scale bar: 50 μm. (*B*) Reg3α-fibrin immunostaining focused on either the top of the preparation (left) or the substratum (right). Reg3α: green; Fibrin: red; DNA: blue. Scale bar: 50 μm. (*C*) Direct immunofluorescence using a FITC-coupled Reg3α (Left). Scale bar: 50 μm. IgG: control using affinity purified IgGs from non-immune rabbit serum (Right). DNA: blue. Scale bar: 100 μm. (*D*) Immunofluorescence of PHHs stressed with H_2_O_2_ and incubated with Reg3α for the indicated duration times. Reg3α: green. DNA: blue. Scale bar: 50 μm.
(TIF)

S2 Fig No intracellular Reg3α was detected in Reg3α-treated primary human hepatocytes. Anti-fibrinogen and anti-Reg3α immunoblotting of the DOC-soluble and insoluble fractions of Reg3α-treated PHHs cultured either without (basal) or with the proteasome inhibitor MG132.
(TIF)

S3 Fig Reg3α and fibrin displayed comparable electrophoretic mobility in the insoluble fractions. Anti-fibrinogen and anti-Reg3α immunoblotting of the DOC-soluble and insoluble fractions of Reg3α-treated PHHs. Proteins were resolved on a 12% native discontinuous acrylamide gel. Asterisks: bands revealing a co-migration of Reg3α and fibrinogen.
(TIF)

S4 Fig Heparin does not inhibit the cytoprotective effects of Reg3α. Left: Immunofluorescence of PHHs exposed to TNFα/ActD, Reg3α and heparin 48h after platting and then incubated for 20h. Reg3α: green. Fibrin: red. DNA: blue. Scale bar: 50 μm. Right: Normalized cell viability.
(TIF)

S5 Fig Reg3α binds the extracellular fibrin network of primary murine hepatocytes. Primary murine hepatocytes (PMHs) were incubated with 0.24 μM of a recombinant Reg3α for 6h either without or with 50 μg/mL of heparin. Control cells were treated with an equivalent volume of vehicle. (*A*) Anti-Reg3α and anti-fibrin immunofluorescence. Fibrin: red; Reg3α: green; DNA: blue. Scale bar: 50 μm. (*B*) Anti-Reg3α and anti-fibrin immunoblotting of DOC-soluble and insoluble fractions.
(TIF)

S6 Fig Activation of the murine Reg3 gene during Fas-induced ALF. (A) Immunofluorescence of liver cryosections from mice intoxicated with the anti-CD95 antibody (Fas) for the indicated duration times and injected with vehicle 2 hours before sacrifice. Fibrin: red; Reg3α: green; DNA: blue. Control: Healthy livers. Scale bar: 50 μm. (B) qPCR for the endogenous Reg3β gene, the murine homolog of Reg3α, in ALF-bearing mice at the indicated times post-Fas.
(TIF)

S7 Fig Practically no Reg3α was accumulated in liver DOC-soluble fractions of treated mice. Anti-Reg3α and anti-fibrinogen immunoblotting in DOC-soluble fractions of liver extracts from mice intoxicated with the anti-CD95 antibody (Fas) and injected with Reg3α or vehicle at different times post-Fas.
(TIF)

S8 Fig Fibrin deposition in liver of patients with chronic and malignant disorder. Anti-fibrin (red staining) immunofluorescence of cryosections of liver explants from patients with different liver disorders. A: adjacent non-neoplastic cirrhotic tissue; B: non-neoplastic parenchyma adjacent of gallbladder carcinoma; C: non-neoplastic parenchyma adjacent of colon cancer metastasis; D: Fibrosis stage F3; E and F: Cirrhosis stage F4. Scale bars: 50 μm.
(TIF)
