# Supplementary figures and images for: The Reg3α (HIP/PAP) Lectin Suppresses Extracellular Oxidative Stress in a Murine Model of Acute Liver Failure
Source: PLoS One. 2015 May 4;10(5):e0125584. doi: 10.1371/journal.pone.0125584 (PMC4418718; doi:10.1371/journal.pone.0125584)

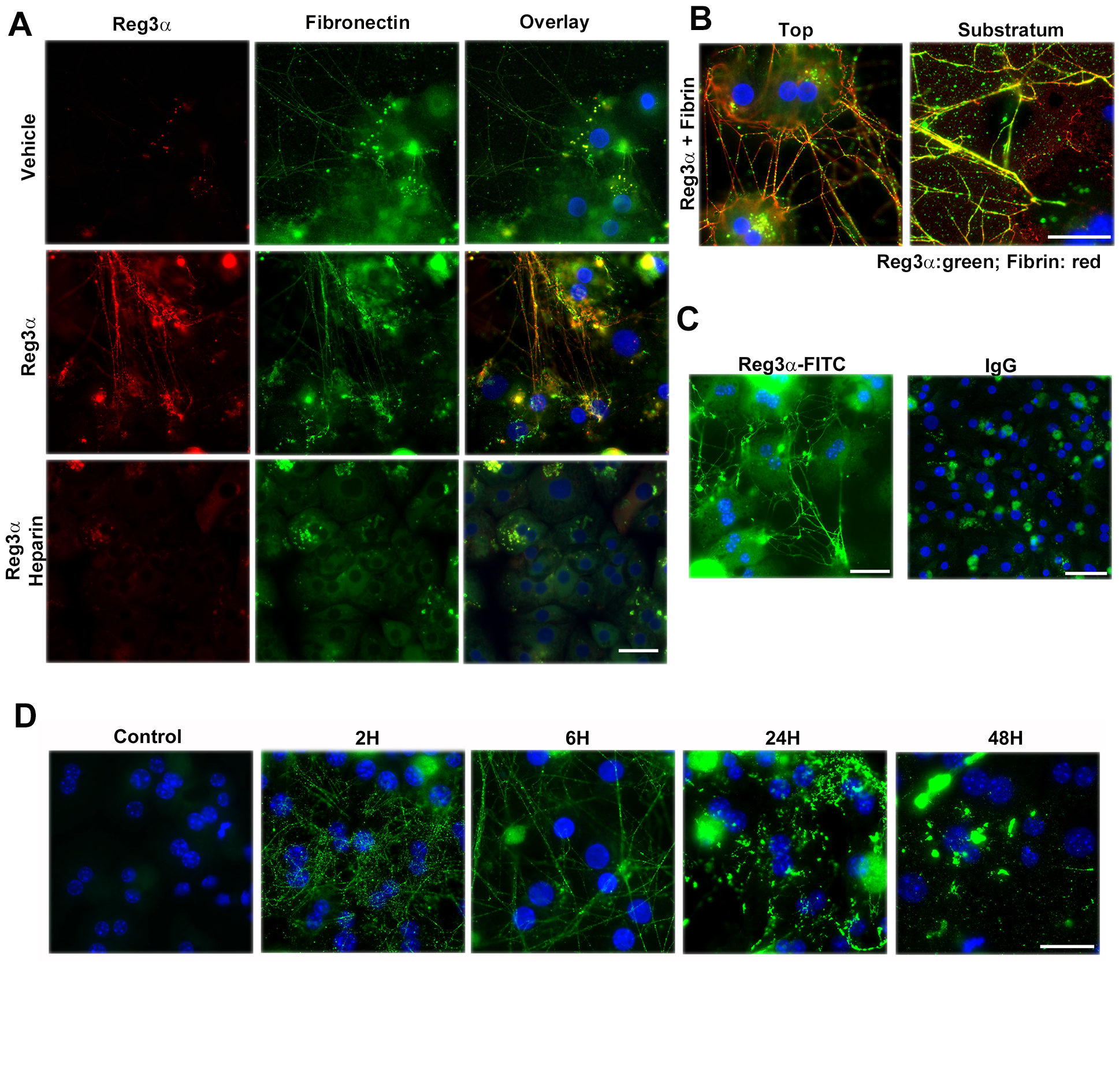

Supplement: S1 Fig — (A-C) Primary human hepatocytes (PHHs) were incubated with 0.24 μM of a recombinant Reg3α for 6h either without or with 50 μg/mL of heparin. Control cells were treated with an equivalent volume of vehicle. (A) Immunofluorescence of Reg3α-treated PHHs either without (middle) or with heparin (bottom). Reg3α: red; Fibrinonectin: green; DNA: blue. Scale bar: 50 μm. (B) Reg3α-fibrin immunostaining focused on either the top of the preparation (left) or the substratum (right). Reg3α: green; Fibrin: red; DNA: blue. Scale bar: 50 μm. (C) Direct immunofluorescence using a FITC-coupled Reg3α (Left). Scale bar: 50 μm. IgG: control using affinity purified IgGs from non-immune rabbit serum (Right). DNA: blue. Scale bar: 100 μm. (D) Immunofluorescence of PHHs stressed with H2O2 and incubated with Reg3α for the indicated duration times. Reg3α: green. DNA: blue. Scale bar: 50 μm. (TIF) [file pone.0125584.s001.tif]

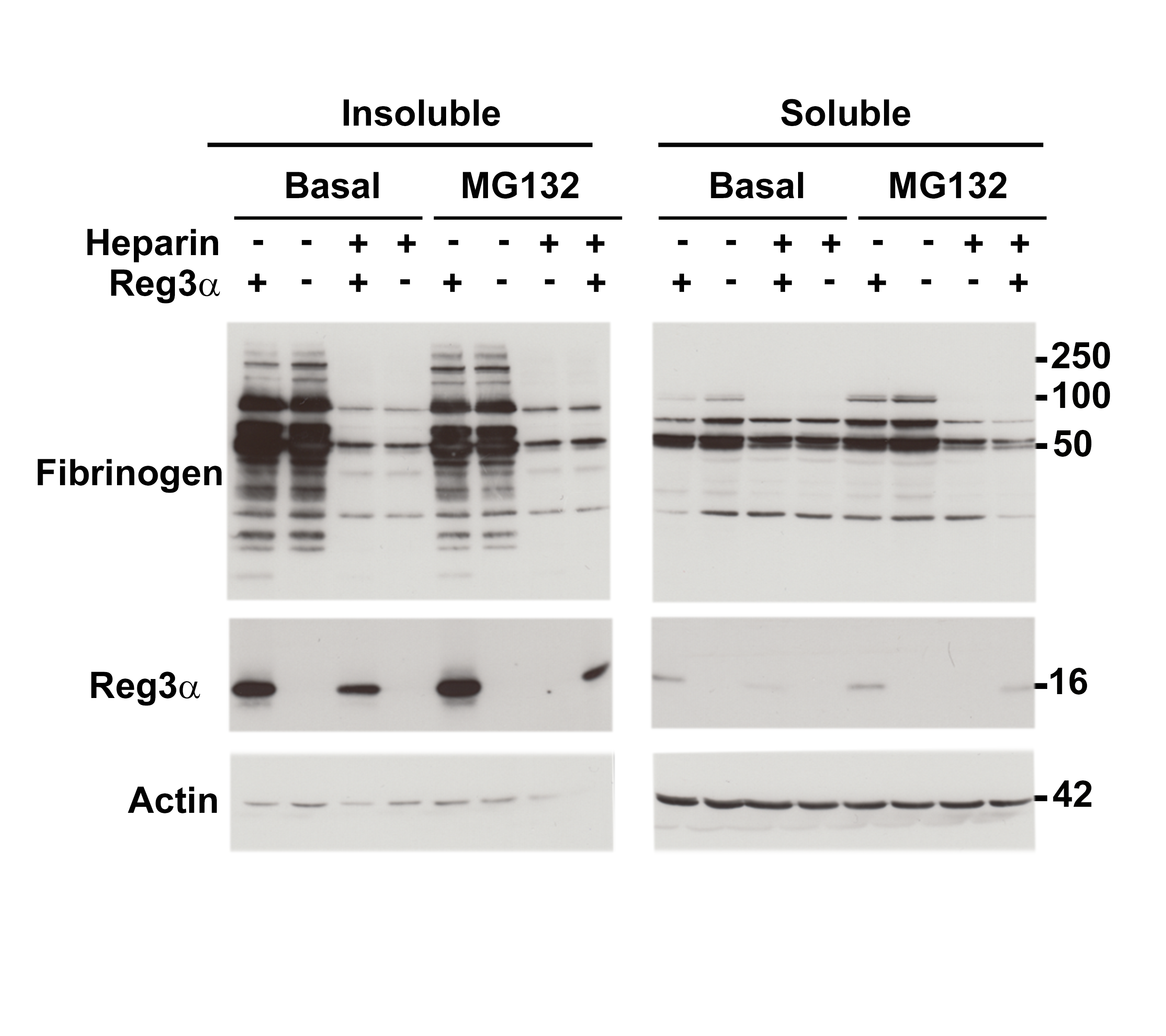

Supplement: S2 Fig — Anti-fibrinogen and anti-Reg3α immunoblotting of the DOC-soluble and insoluble fractions of Reg3α-treated PHHs cultured either without (basal) or with the proteasome inhibitor MG132. (TIF) [file pone.0125584.s002.tif]

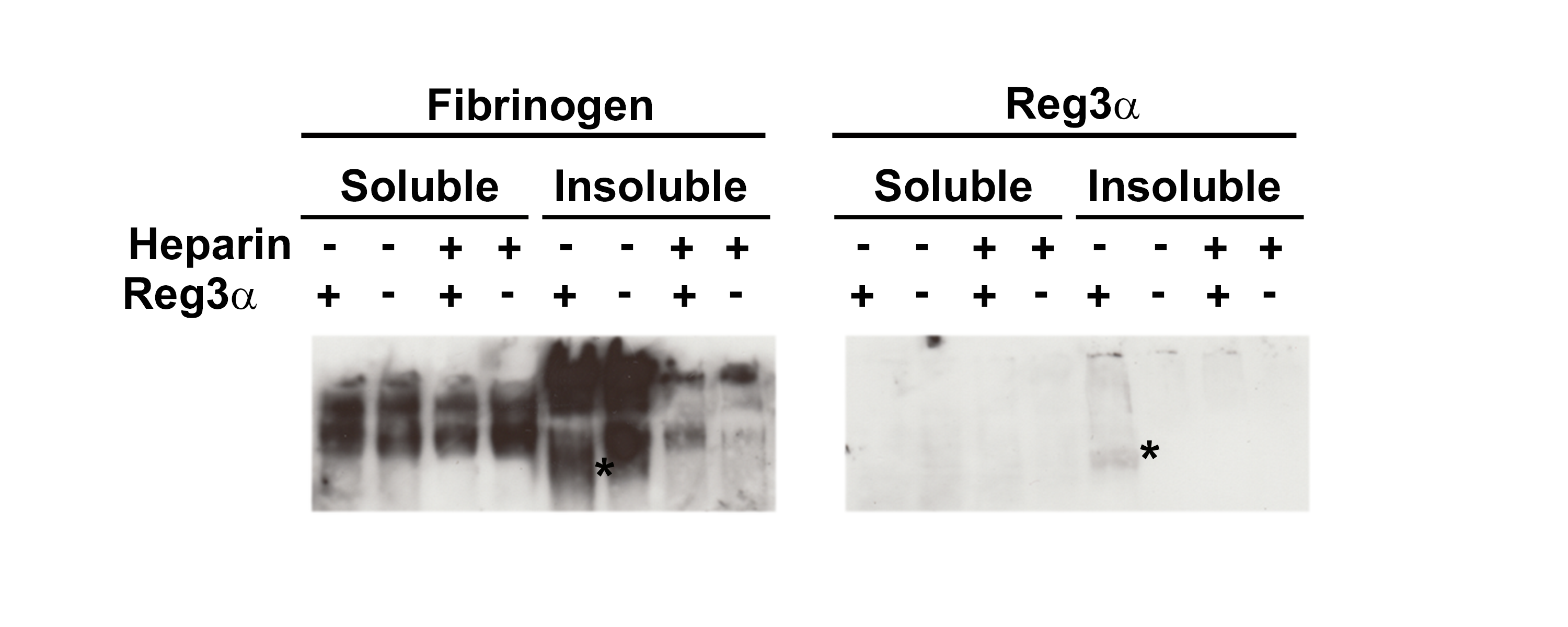

Supplement: S3 Fig — Anti-fibrinogen and anti-Reg3α immunoblotting of the DOC-soluble and insoluble fractions of Reg3α-treated PHHs. Proteins were resolved on a 12% native discontinuous acrylamide gel. Asterisks: bands revealing a co-migration of Reg3α and fibrinogen. (TIF) [file pone.0125584.s003.tif]

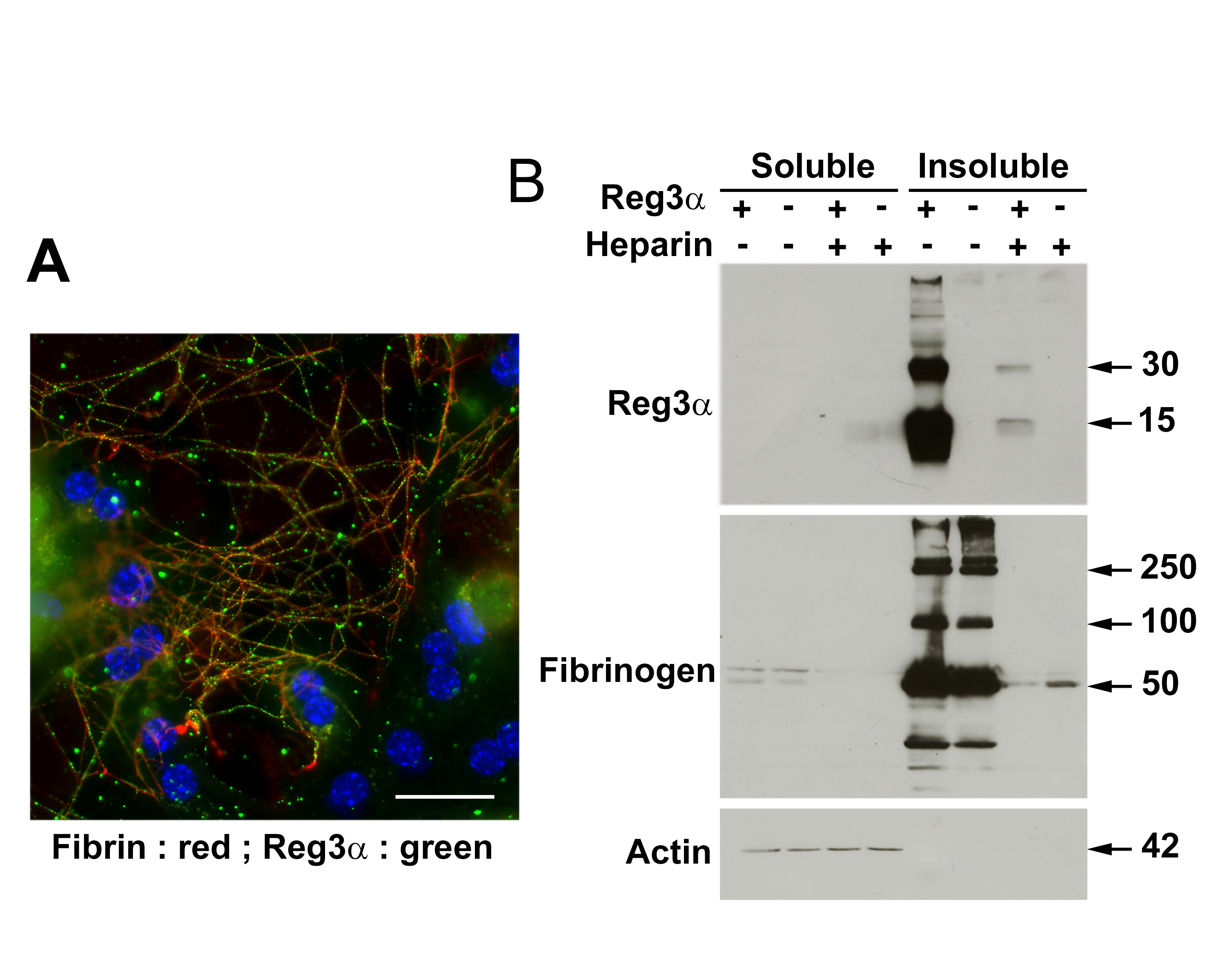

Supplement: S4 Fig — Left: Immunofluorescence of PHHs exposed to TNFα/ActD, Reg3α and heparin 48h after platting and then incubated for 20h. Reg3α: green. Fibrin: red. DNA: blue. Scale bar: 50 μm. Right: Normalized cell viability. (TIF) [file pone.0125584.s004.tif]

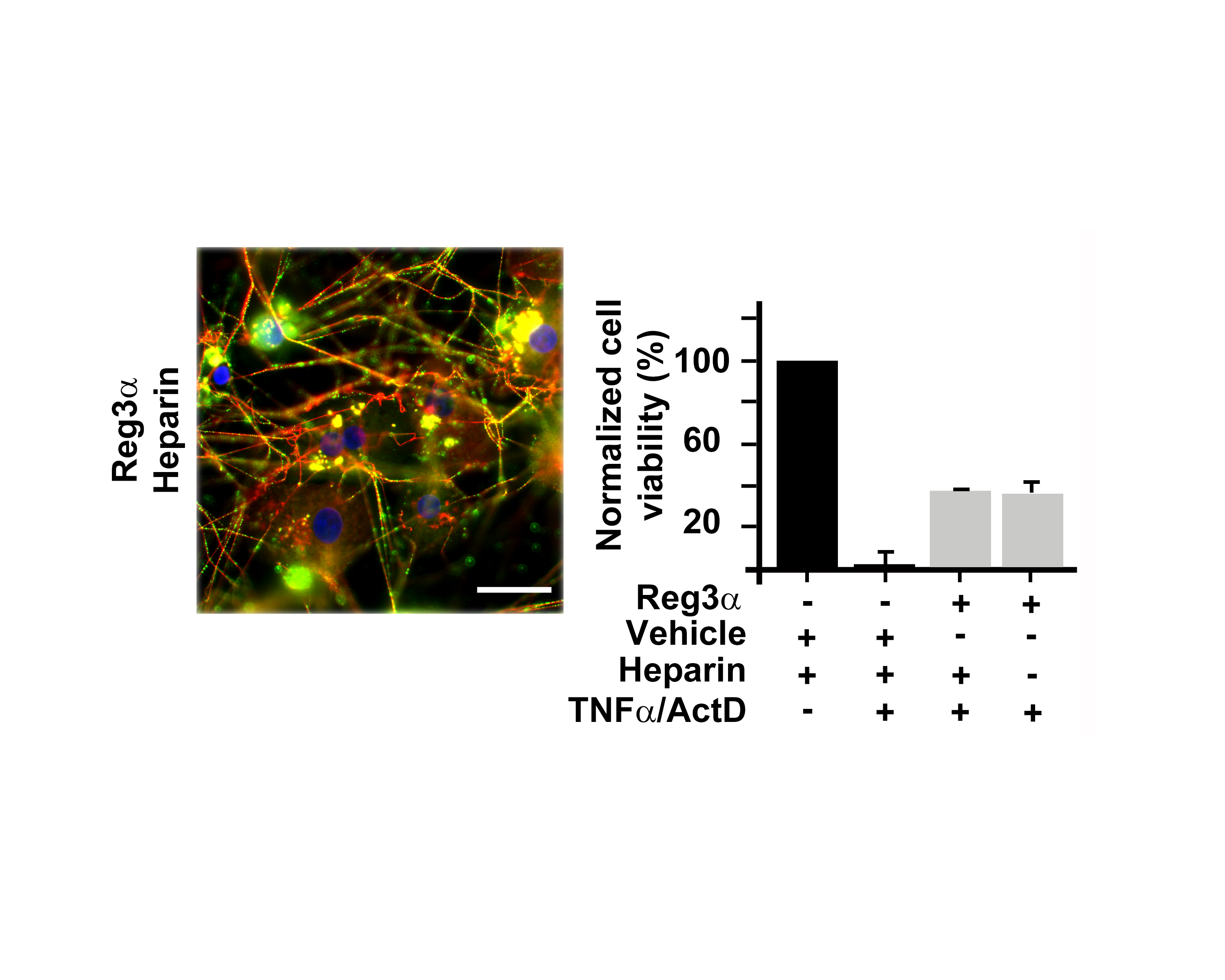

Supplement: S5 Fig — Primary murine hepatocytes (PMHs) were incubated with 0.24 μM of a recombinant Reg3α for 6h either without or with 50 μg/mL of heparin. Control cells were treated with an equivalent volume of vehicle. (A) Anti-Reg3α and anti-fibrin immunofluorescence. Fibrin: red; Reg3α: green; DNA: blue. Scale bar: 50 μm. (B) Anti-Reg3α and anti-fibrin immunoblotting of DOC-soluble and insoluble fractions. (TIF) [file pone.0125584.s005.tif]

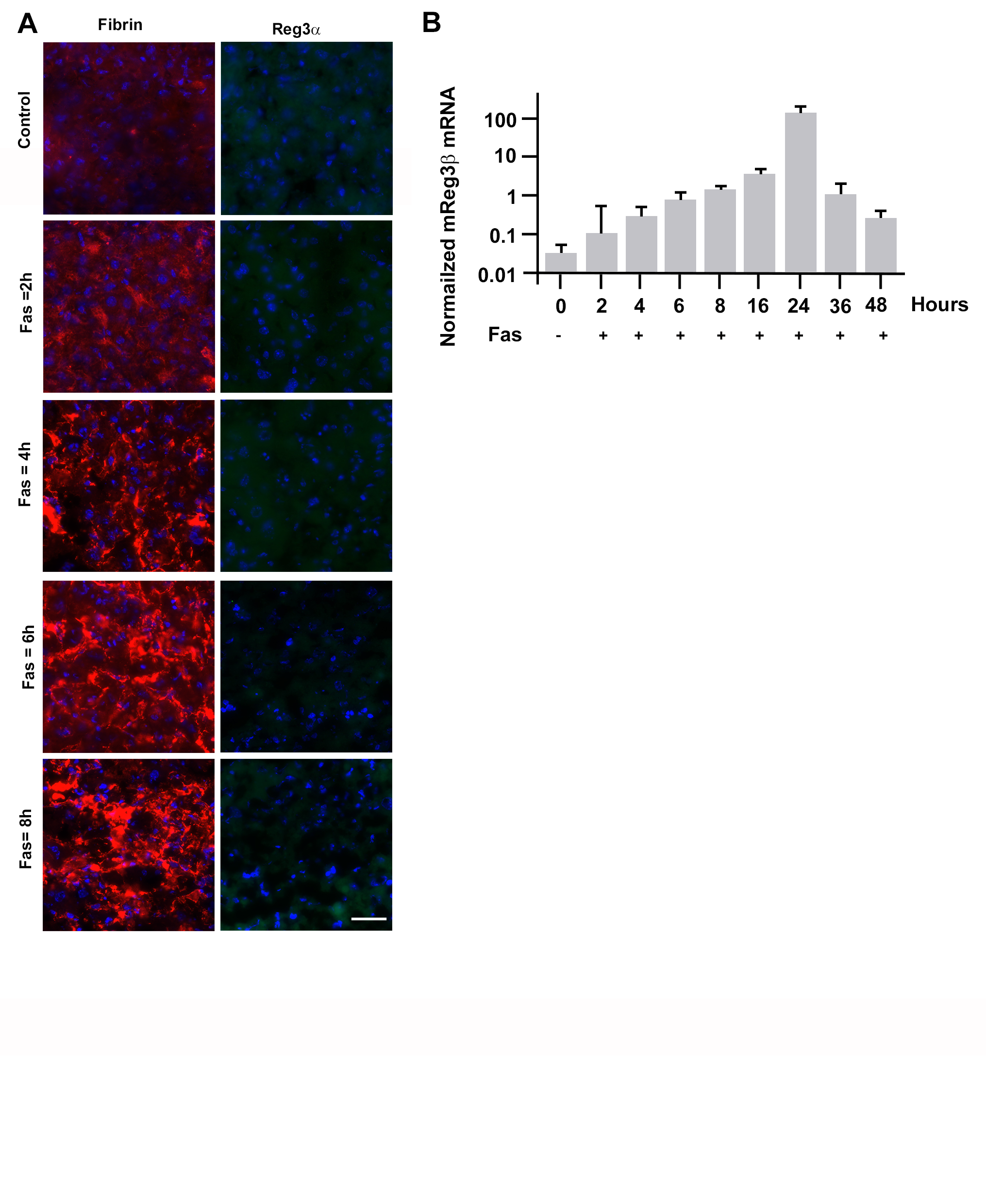

Supplement: S6 Fig — (A) Immunofluorescence of liver cryosections from mice intoxicated with the anti-CD95 antibody (Fas) for the indicated duration times and injected with vehicle 2 hours before sacrifice. Fibrin: red; Reg3α: green; DNA: blue. Control: Healthy livers. Scale bar: 50 μm. (B) qPCR for the endogenous Reg3β gene, the murine homolog of Reg3α, in ALF-bearing mice at the indicated times post-Fas. (TIF) [file pone.0125584.s006.tif]

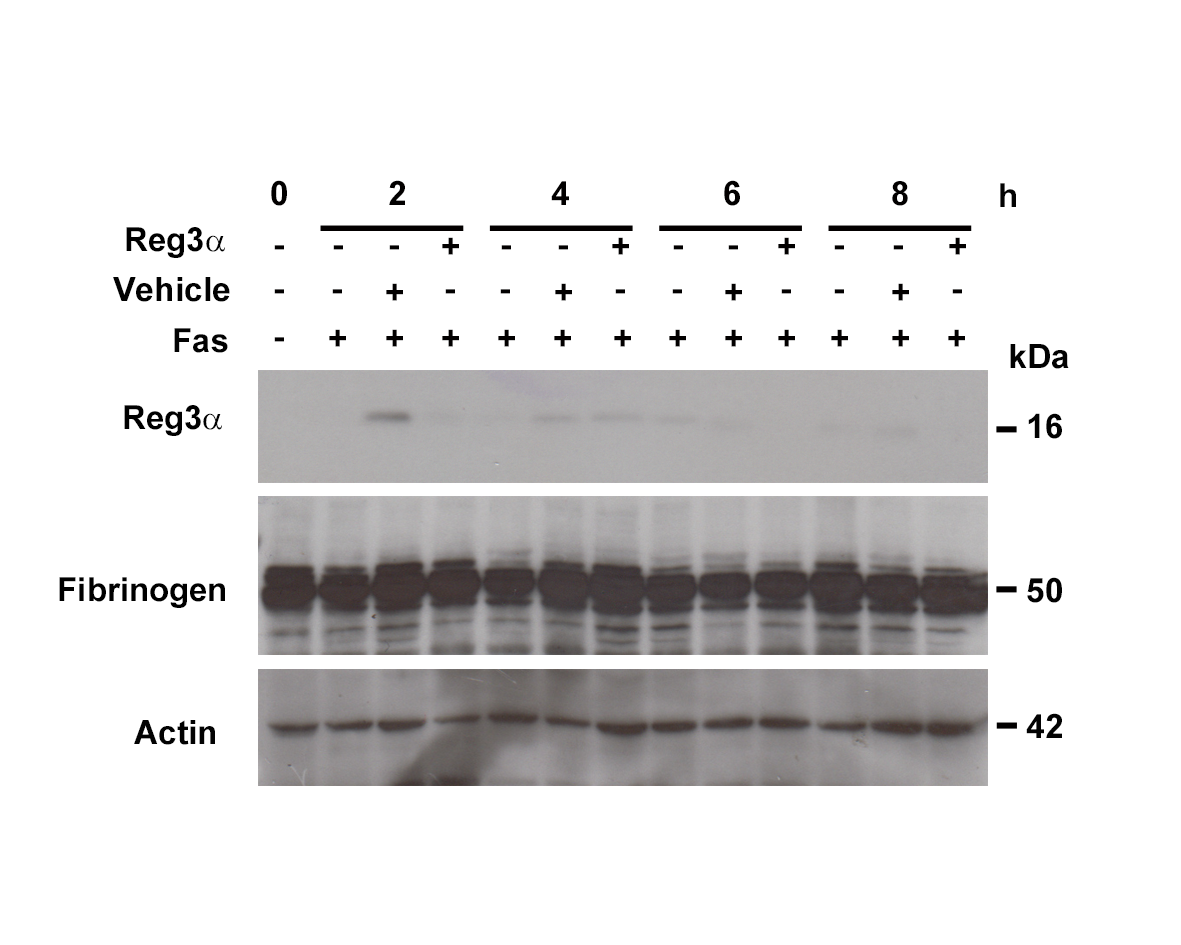

Supplement: S7 Fig — Anti-Reg3α and anti-fibrinogen immunoblotting in DOC-soluble fractions of liver extracts from mice intoxicated with the anti-CD95 antibody (Fas) and injected with Reg3α or vehicle at different times post-Fas. (TIF) [file pone.0125584.s007.tif]

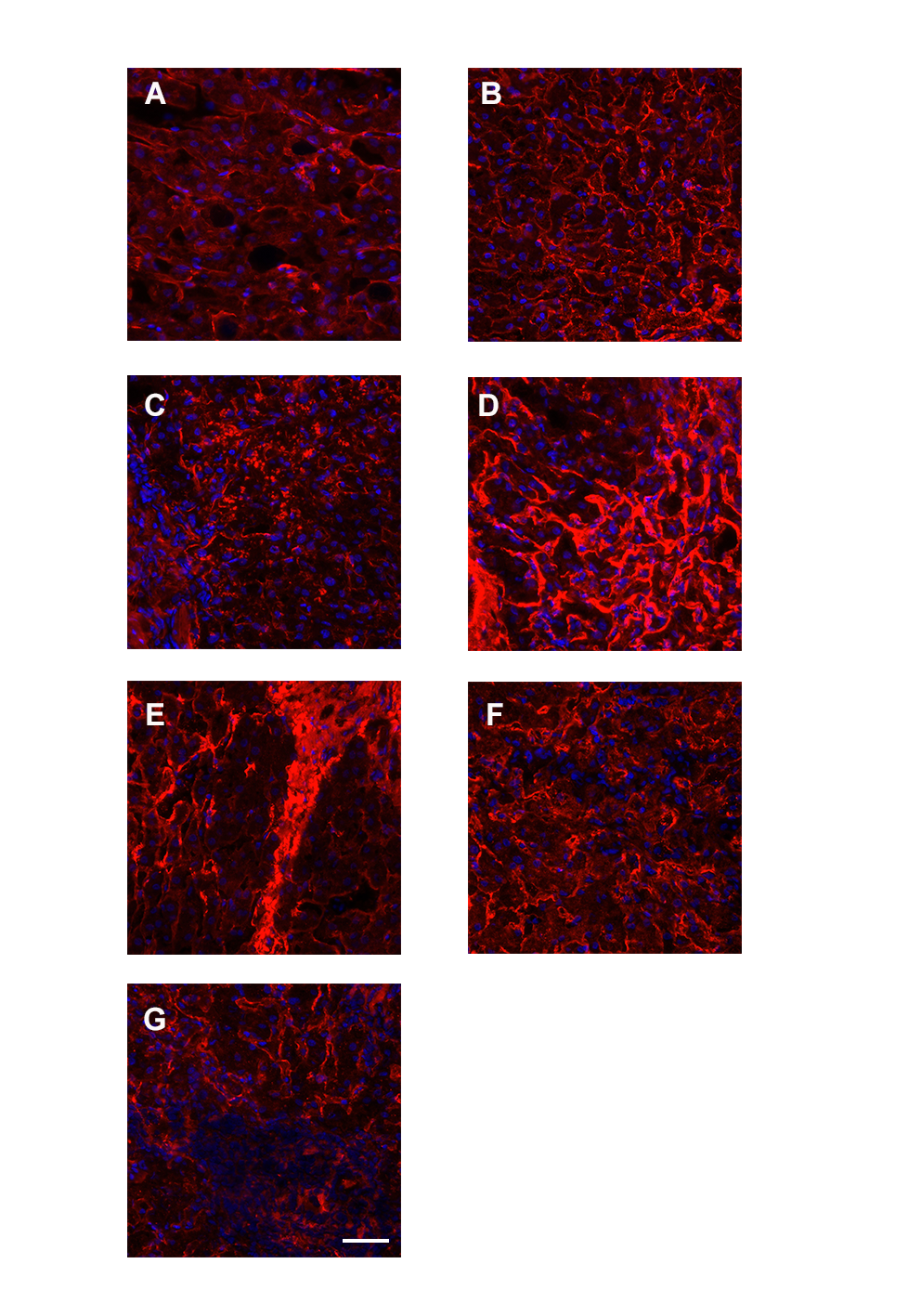

Supplement: S8 Fig — Anti-fibrin (red staining) immunofluorescence of cryosections of liver explants from patients with different liver disorders. A: adjacent non-neoplastic cirrhotic tissue; B: non-neoplastic parenchyma adjacent of gallbladder carcinoma; C: non-neoplastic parenchyma adjacent of colon cancer metastasis; D: Fibrosis stage F3; E and F: Cirrhosis stage F4. Scale bars: 50 μm. (TIF) [file pone.0125584.s008.tif]
